# SLC2A3 is a Potential Factor for Head and Neck Squamous Cancer Development through Tumor Microenvironment Alteration

**DOI:** 10.2174/0115665232291300240509104344

**Published:** 2024-05-21

**Authors:** Wei Jiang, Sheng Xu, Ping Li

**Affiliations:** 1 Guangxi Key Laboratory of Early Prevention and Treatment for Regional High Frequency Tumor, Guangxi Medical University, Nanning, Guangxi Zhuang Autonomous Region, China;; 2 College of Stomatology, Guangxi Medical University, Nanning, Guangxi Zhuang Autonomous Region, China;; 3 Department of Dental Laboratory, Guangxi Medical University College of Stomatology, Nanning, Guangxi Zhuang Autonomous Region, China;; 4 Department of Pathology, Guangxi Medical University College of Stomatology, Nanning, Guangxi Zhuang Autonomous Region, China

**Keywords:** SLC2A3, tumor microenvironment, CD8+ T cell, immune infiltration, head and neck squamous carcinoma, tumor immunity

## Abstract

**Introduction:**

Tumor immunity has garnered increasing attention in cancer treatment and progression. However, there is still a challenge in understanding the mechanisms of specific molecules affecting the clinical prognosis and tumor microenvironment (TME).

**Methods:**

Here, we applied the ESTIMATE algorithm to calculate the immune and stromal scores in 504 HNSC cases from TCGA. Patients were grouped according to the median value of the immune and stromal. Clinicopathological characteristics and differentially expressed genes (DEG) were analyzed. Subsequently, LASSO, COX regression, survival analysis, and clinicopathological characteristics were conducted. Subsequently, SLC2A3 was determined as a predictive factor that high expression of SLC2A3 at the mRNA and protein levels predicted a worse clinical prognosis. GSEA25099 was utilized for external validation of immune infiltration, while tissue PCR, IHC, and Western Blot were used to confirm the expression levels of SLC2A3.

**Results:**

A series of immune-infiltration analyses showed that SLC2A3 expression was negatively correlated with CD8+ T cells, significantly affecting the survival prognosis of HNSC. In the GSEA analysis, the high expression of SLC2A3 was mainly enriched for immune-related biological processes. Meanwhile, high expression of SLC2A3 possessed higher TIDE scores and was also strongly positively correlated with a series of immune checkpoints affecting survival prognosis, thus causing greater susceptibility to immune escape.

**Conclusion:**

Conclusively, SLC2A3 is a potential oncogene and factor of HNSC development, notably by an altered state of the immune microenvironment, immune-suppressive regulation, and immune escape.

## 1 INTRODUCTION

Head and neck tumors rank sixth in terms of prevalence as one of the most common types of malignant tumors worldwide. It originates from the mucosal epithelial cells of the upper respiratory and digestive tract, including squamous cell carcinoma of the oral cavity, larynx, laryngopharynx, and oropharynx. Although there have been significant advancements in the treatment for head and neck squamous cell carcinoma (HNSC) in recent decades, the prognosis for this disease remains unfavorable, with a 5-year survival rate ranging from 50% to 60% [1, 2]. Thus, studying the underlying molecular mechanism of HNSC may facilitate the development of molecularly targeted therapies for this disease.

TME, or the tumor microenvironment, comprises different types of cells, including tumor cells, stromal cells, and immune cells [3-5]. The presence of non-tumor elements in the tumor microenvironment, including immune cells and stromal cells, can potentially impact the development and prognosis of the tumor [6, 7]. Extensive research has examined the influence of immune cells within the TME on tumor development and advancement. Among these immune cells, CD8+T cells have been extensively sampled and proven to have the most significant impact on the survival prognosis of HNSC [8, 9]. Mechanistically, CD8+ T cells secret large amounts of IFN-γ and the protease granzyme B, which act synergistically to kill infected or tumor cells [8, 10]. Clinically, the density and distribution of CD8+ T cells at the tumor infiltration margin are significantly and positively correlated with survival prognosis [11, 12]. Beyond immune cells, the tumor stroma, characterized by a modified extracellular matrix produced by all TME cell types, establishes a complex fiber network. This network endows mechanical strength, proteoglycans, growth factors, and cytokine-binding capabilities, all of which are essential for facilitating tumor invasion and metastasis [4, 13, 14]. For instance, immunosuppressive cytokines like interleukin-10 and transforming growth factor-β secreted by tumor-associated macrophages (TAMs) promote tumor growth and are associated with poorer outcomes [15-19]. Due to these cooperative interactions, malignant phenotypes like immortal proliferation, resistance to apoptosis, and resistance to immunity are potentially influenced by the peri-cancer cells in TME [20-25]. Despite these findings, the underlying mechanisms are yet to be fully comprehended.

To explore this unknown, the ESTIMATE (Estimation of Stromal and Immune cells in Malignant Tumor tissues using Expression data) algorithm has been employed to assess the stromal and immune composition within tumors [26-32]. This study leverages ESTIMATE, alongside multiple analyses, to identify a significant TME biomarker, solute carrier family two member 3 (SLC2A3). SLC2A3 facilitates glucose transport across cell membranes intracellularly, essential for glycolysis. This function supports the increased energy needs of rapidly proliferating tumor cells, leading to enhanced metabolism and glucose intake [33-36]. Elevated SLC2A3 expression has been reported to correlate with adverse outcomes in tongue cancer, influences the epithelial-mesenchymal transition (EMT) in colorectal cancer, and is linked to detrimental TME changes, such as macrophage infiltration, in gastric and breast cancers [37-40]. These findings underscored the importance of further research into SLC2A3’s prognostic implication and its association with immune infiltration in various cancers.

In the current research, by comparing the high and low scores of immune and stromal components in HNSC samples, we analyzed by computing differentially expressed genes (DEGs), regression, and survival analysis. Consequently, this comprehensive analysis identified SLC2A3 as a key gene associated with alternation in the TME and prognostic significance. Immune infiltration was validated using the Gene Expression Omnibus (GEO) database. To validate the RNA and protein expression levels, tissue PCR, IHC, and Western blot techniques were employed.

## 2 MATERIALS AND METHODS

### 2.1 Data Source and Tissue Samples

Gene expression raw data and clinical information of 504 HNSC patients were obtained from the TCGA database (https://portal.gdc.cancer.gov/). Clinical data included age, prognostic stage, TNM stage, tumor grade, survival status, lymphovascular invasion, and perineural invasion. Protein expression analysis was performed using the CPTAC database in UALCAN (http://ualcan.path.uab.edu/analysis-prot.html), while immunohistochemistry image data were obtained from the HPA (https://www.proteinatlas.org/). For validation, raw transcriptome profiling data of GSE25099 were acquired from the GEO database. Additionally, ten pairs of HNSC tissues and adjacent tissues were collected from the First Affiliated Hospital of Guangxi Medical University with informed consent and ethical approval (No.2023-K064-01).

### 2.2 TME Analysis *via* ESTIMATE Algorithm

The ESTIMATE algorithm, implemented in R language version 3.5.1 with the ESTIMATE package, was utilized to calculate the immune and stromal cell infiltration scores in the tumor microenvironment. These scores determine the fractions of stromal cells and immune cells based on gene expression characteristics. The immune and stromal scores of the tumor samples from the 504 HNSC cases in this study were computed using the ESTIMATE algorithm.

### 2.3 Differentially Expressed Genes

Five hundred-four tumor samples were divided into high-score and low-score groups based on immune score (IS) and stromal score (SS) medians. The high-score group included samples with high IS and SS, while the low-score group included samples with low IS and SS. Differential gene expression analysis was conducted using R version 3.5.1 with DESq2 package. Genes were considered differentially expressed when |Log2foldchange| > 1.5 and FDR < 0.05.

### 2.4 Regression Analysis and Survival Analysis

LASSO and multiple COX regression analyses were used to explore the association between gene expression levels and overall survival (OS). R software was utilized to integrate survival data and gene expression, applying Lasso regression analysis. Multiple COX regression analysis was then performed to identify clinically significant high-risk genes significantly affecting survival prognosis (*p*-value < 0.05). Validation of these findings was performed using the GEPIA2021 online database (https://gepia2021.cancer-pku.cn), based on the TCGA database, for survival analyses.

### 2.5 Immune Infiltration and Immune Escape Analysis

Genes identified from LASSO and multiple COX regression analyses underwent immune infiltration assays: CIBERSORT, ssGSEA, EPIC, and ESTIMATE. CIBERSORT estimated immune cell proportions in the tumor microenvironment (TME) based on transcriptome data, while ssGSEA calculated the infiltration degree of various immune cell types. The EPIC algorithm analyzed the correlation between gene expression and immune cells.

To assess immune cell infiltration and conduct survival analysis, we utilized online tools GEPIA2021 and TIMER (https://cistrome.shinyapps.io/timer/) using RNA seq expression profiles from the TCGA database.

Immune checkpoints and the TIDE algorithm were evaluated using the IMMUNITY module in the aclbi online tool. The TIDE score assessed tumor immune escape mechanisms, including dysfunctional tumor-infiltrating cytotoxic T lymphocytes (CTL) and rejection of CTL by immunosuppressive factors. Immune checkpoints, such as PD1, CTLA4, T cell activation agonists, TIGIT, CAR-T, and others were examined in this study.

### 2.6 Gene Set Enrichment Analysis

GSEA was performed on 504 HNSC datasets using GSEA 4.2.3 Software, utilizing the “h.all.v6.1.symbols.gmt” gene set. The HNSC cases were divided into low and high groups based on the median mRNA expression of SLC2A3, with 1000 gene set permutations. Enrichment was deemed significant when the false discovery rate (FDR) score was below 25%, and the *p*-value was less than 0.05.

### 2.7 Validation in Independent Datasets

To verify the correlation between SLC2A3 and screened immune cells in expression, we selected the tissue microarray sequencing GSE25099, which contained transcriptome profiling data of 57 HNSC and 20 normal samples.

### 2.8 Western Blot and Antibodies

Equal amounts of protein were separated by electrophoresis on a 4-12% SDS-PAGE gel and subsequently transferred to a nitrocellulose membrane (Thermo Fisher Scientific, USA) with 5mg Protein ladder (26619/26620, Thermo Fisher Scientific, USA). The membrane was incubated with primary antibodies, including SLC2A3 (1:10000, Santa Cruz, USA) and GAPDH (1:10000, Santa Cruz, USA), overnight at 4°C. Peroxidase-conjugated secondary antibodies were added and incubated for 2 hours at room temperature. The fluorescent signals were then detected using the Bio-rad ChemiDoc MP system (Bio-rad, CA, USA).

### 2.9 Real-Time Quantitative PCR

First-strand complementary DNA was created using a First-Strand Reverse Transcription System (Transgene, Beijing, China). The real-time RT-PCR was then conducted on the cDNA samples, using the SYBR Green PCR master mix and the Step One Plus System from Applied Biosystems (USA). The primer sequences were as follows: SLC2A3-F,5′-CCUGAGAAGAUCAUAAAGGAATT-3'; SLC2A3-R, 5′-UUCCUUUAUGAUCUUCUCAGGTT-3′; GAPDH-F, 5′-CGGATTTGGTCGTATTGGG-3'; GAPDH-R, 5′-CTGGAAGATGGTGATGGGATT-3′.

### 2.10 Immunohistochemical Staining

After deparaffinization and rehydration, antigen retrieval was performed using 5% urea buffer heated in a microwave for 5 minutes. Endogenous peroxidase activity was blocked by incubating the tissue in 3% H_2_O_2_ for 30 minutes. Tissue sections were then incubated with the primary antibody overnight at 4°C, followed by incubation with a secondary antibody for 1 hour at room temperature. The peroxidase reaction was visualized using a 3,3'-diaminobenzidine (DAB) reagent for staining, and hematoxylin was used for counterstaining. Microscopic images were captured using an Olympus C-5050 microscope (Japan).

### 2.11 Statistical Analysis

Statistical analyses were performed using R(4.0.2) and spss22.0 software. Correlation analysis used Spearman’s coefficient. Survival curves were constructed using the Kaplan-Meier method (log-rank test). Student's t-test was used for the analysis of variance in two groups. Multiple COX regression analyses identified independent predictors of survival. Significance levels were denoted as * for *p* < 0.05, ** for *p* < 0.01, *** for *p* < 0.001, and ns for no significance.

## 3 RESULTS

## 3.1 Analysis Workflow of this Study

The data processing in this paper is shown in Fig. (**1**). Using the ESTIMATE algorithm, we evaluated the ImmuneScore and StromalScore of 504 patients with HNSC in the TCGA database. Patients were grouped according to median score (Stromal Score high, Stromal Score low, Immune Score high, Immune Score low). Clinicopathological characteristics and differentially expressed genes (DEG) were analyzed. The intersections of 1301 upregulated genes and 120 downregulated were subsequently performed LASSO, multi-COX regression, and survival analysis, resulting in SLC2A3. A series of functional enrichment and immune-related analyses were conducted on SLC2A3.

## 3.2 Higher ImmuneScore and StromalScore are Potential Implications of Worse Clinical Outcomes

**Fig. (2):**
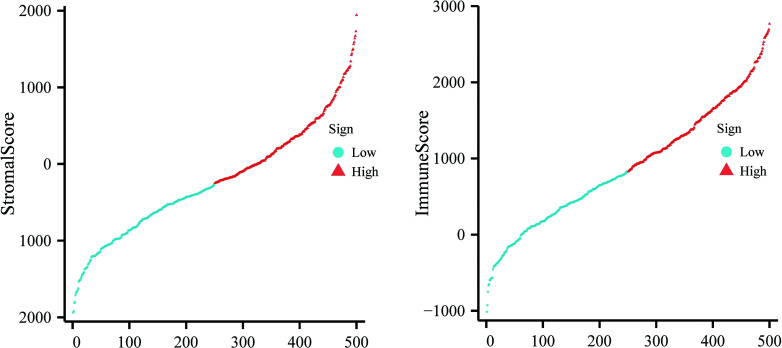
504 HNSC cases were divided into high and low groups according to the median of stromal and immune scores.

We used the ESTIMATE algorithm to investigate the composition and infiltration of stromal and immune cells (Fig. **2**). Then, we performed a series of clinicopathologic feature groupings to evaluate and assign clinical value to the ESTIMATE algorithm. As shown in Table **1**, patients with M1 in the M stage had higher Stromalscore. Moreover, patients with III+IV in Tumor grade, advanced stage in Clinical stage, and higher T stage had higher Immune scores. These results suggest that the degree of infiltration of stromal and immune cells is, to some extent, related to the worse clinical outcomes of patients.

## 3.3 DEGs Shared by ImmuneScore and StromalScore were Mainly Enriched in Immune-related Biological Processes

**Fig. (3):**
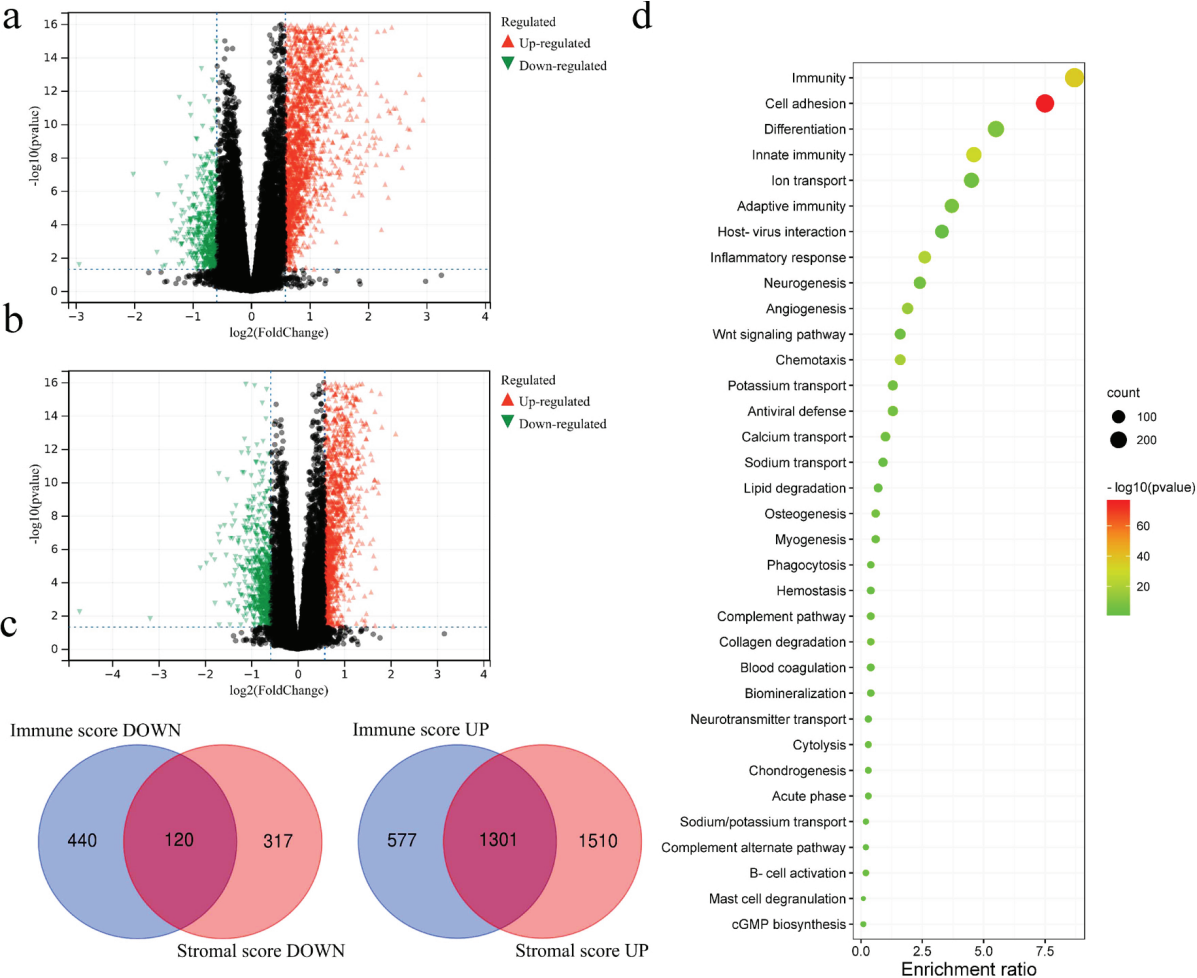
Volcano map of DEGs for stromalscore (**a**) and immunescore (**b**), |FC| > 1.5 and *p* < 0.05 were set as cutoff; (**c**) The intersected genes of Immunescore and Stromalscore; (**d**) GO enrichment analysis.

We conducted a differential gene expression analysis to investigate the specific changes in gene expression within the TME in relation to immune and stromal components. Figs. (**3a** and **3b**) illustrate the results of this analysis, showing the alterations in gene expression between samples with high and low immune and stromal scores. A total of 2439 differentially expressed genes (DEGs) were identified in samples with high ImmuneScore compared to those with low scores, with 1878 genes up-regulated, and 560 genes downregulated. Similarly, 3249 DEGs were found in samples with high StromalScore compared to those with low scores, including 2811 up-regulated and 437 down-regulated genes. Interestingly, 1301 genes were co-upregulated, while 120 genes were co-downregulated in both immune and stromal components (Fig. **3c**). GO enrichment analysis revealed that these differentially expressed genes were predominantly involved in immune-related biological processes, such as immunity, innate immunity, adaptive immunity, and inflammatory response (Fig. **3d**). These findings suggest that immune-related molecules and cells are crucial role in shaping the tumor microenvironment.

## 3.4 Among the Genes Regulated by Tumor Microenvironment, SLC2A3 May Affect the Clinical Prognosis

**Fig. (4):**
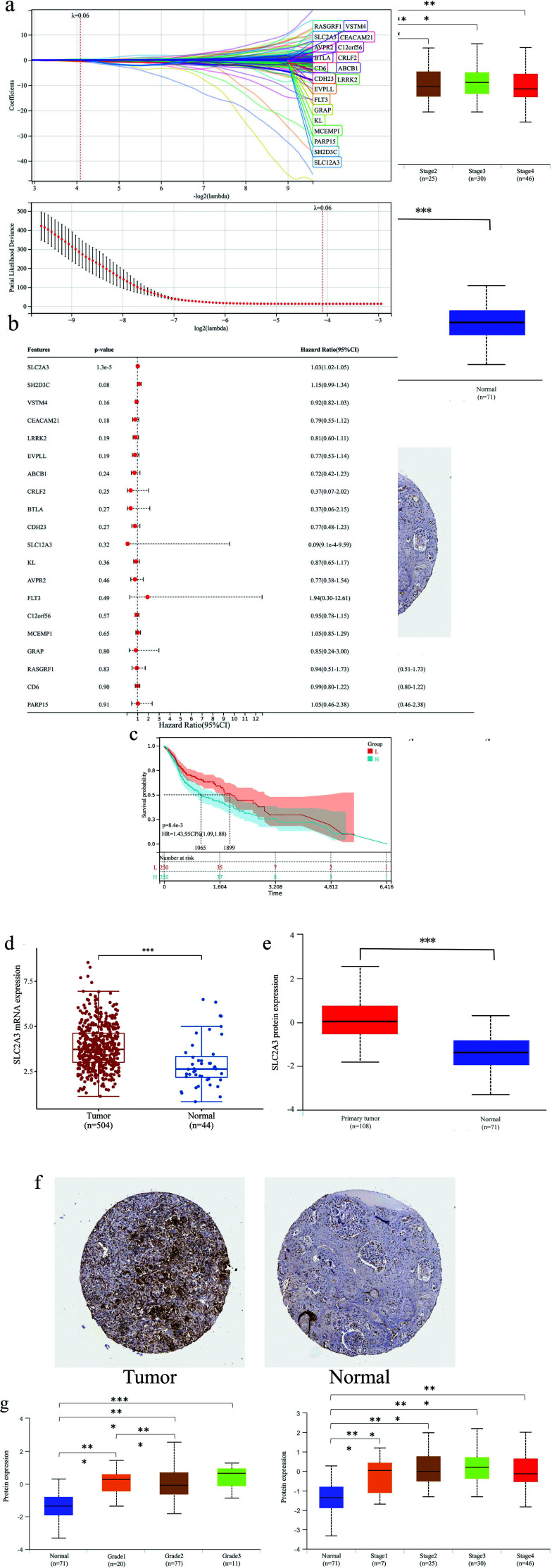
Lasso-COX regression analysis. (**a**) lasso regression with Lamda value = 0.05 and 10-fold cross-validation. (**b**) forest plot of multiple COX regression. (**c**) Kaplan-Meier OS curves of SLC2A3. (**d-f**) SLC2A3 expression of mRNA (**d**, data from TCGA database) and protein (**e**, data from CPTAC database) in tumor tissues and normal tissues. (**f**) IHC results of HNSC tissue (left) and normal tissue (right). (**g**) The relationship between the expression level of SLC2A3 protein and Tumor grade (left) and Clinical stage (right), respectively. Significance levels were denoted as * for *p* < 0.05, ** for *p* < 0.01, *** for *p* < 0.001, and ns for no significance.

We took the common DEGs derived from the last step, consisting of 1301 up-regulated genes and 120 down-regulated genes. By integrating survival time, survival status, and gene expression data, we analyzed using LASSO and multiple COX regression methods. Consequently, 20 candidate genes were obtained, and finally, the forest plot showed a significant effect of SLC2A3 on survival prognosis (Figs. **4a** and **b**). The Kaplan-Meier OS curves of the high- and low- groups, according to the median expression of SLC2A3, were significantly different in median OS (1065d *vs*. 1899d, *p*=0.0084; Fig. **4c**).

We further investigated the mRNA and protein expression of SLC2A3 on clinical characteristics. We found that SLC2A3 was significantly higher in both protein and mRNA levels in HNSC tumor tissues than in normal tissues (Figs. **4d-f**). Dead patients and patients with higher T, N stage, clinical stage, and tumor grade had higher SLC2A3 mRNA expression (Table **2**). Similarly, in SLC2A3 protein expression, higher clinical stage and tumor grade had significantly higher SLC2A3 expression (Fig. **4g**). These results showed that the high expression of SLC2A3 at the mRNA and protein levels may have worse clinical outcomes.

## 3.5 SLC2A3 Potentially Affects Immune Infiltration and Negatively Correlates with CD8+ T cells

**Fig. (5):**
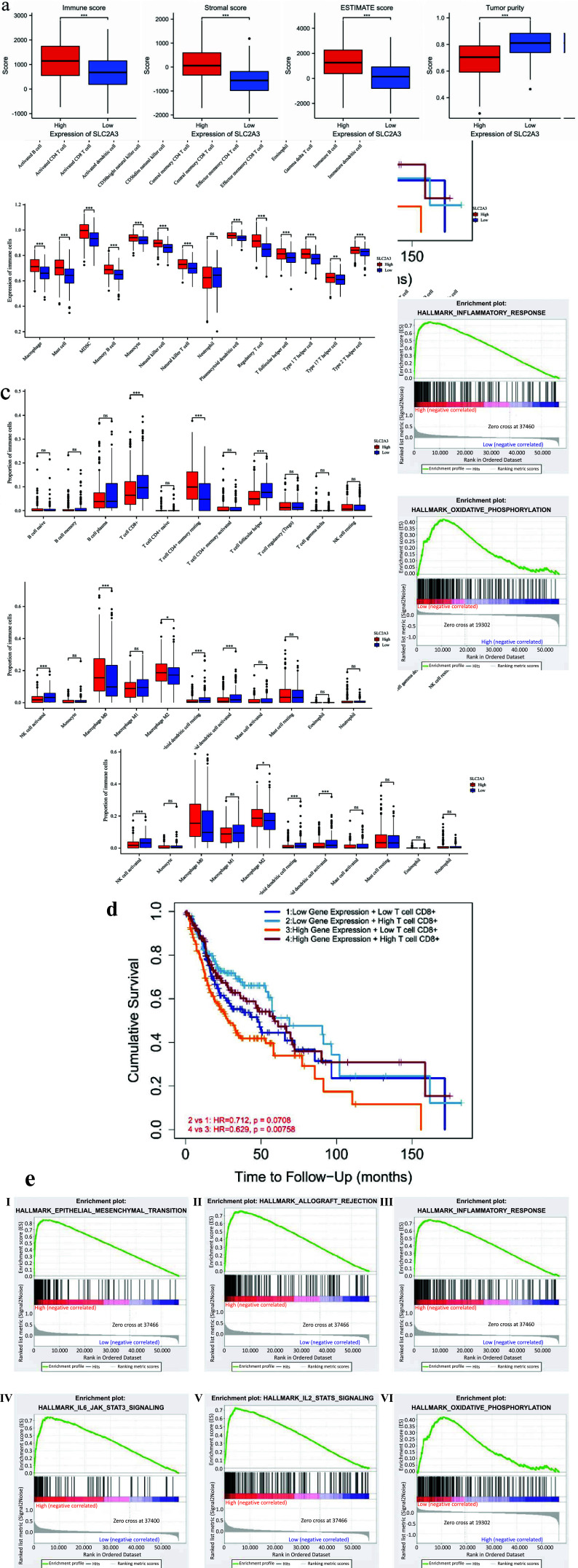
(**a**) ESTIMATE assays obtain immune, stromal, ESTIMATE score,and tumor purity. (**b**) ssGSEA: The box plot of the expression of several types of immune cells. (**c**) the box plot of the proportion of immune cells. (**d**) Cumulative survival analysis of CD8+ T cell x SLC2A3 expression, data from TIMER. (**e**) All six enrichment items in GSEA were statistically significant. The high SLC2A3 expression group was enriched in I-V, ranked by *p*-value from small to large. VI is the enrichment of the low SLC2A3 expression group. Significance levels were denoted as * for *p* < 0.05, ** for *p* < 0.01, *** for *p* < 0.001, and ns for no significance.

Based on the median SLC2A3 mRNA expression, we divided 504 HNSC cases into high- and low-expression groups. We then conducted evaluations using ESTIMATE (Fig. **5a**), ssGSEA (Fig. **5b**), and CIBERSORT (Fig. **5c**). In ESTIMATE, the high expression group of SLC2A3 showed higher scores for stromal cell and immune cell infiltration, as well as lower tumor purity. This analysis suggests that the SLC2A3 high-expression group has a more complex tumor microenvironment and a greater potential regulation.

In the intersection of ssGSEA and CIBERSORT analysis, we observed significant overexpression of CD4+ memory resting, Myeloid Dentri activated, Myeloid Dentri resting, Macrophage M0, Macrophage M2, and T helper in the SLC2A3 high expression group. In contrast, the high expression of SLC2A3 showed a negative association with the expression of CD8+T cells and NK cells. Survival analysis using GEPIA2021 and TIMER revealed that only CD8+ T cell expression significantly influenced the survival of HNSC patients (Fig. **5d**, **S1**). This indicates that high expression of SLC2A3 may lead to low expression of CD8+T cells, resulting in poorer survival.

GSEA analysis showed that the high expression of SLC2A3 was predominantly enriched in immune-related pathways, such as allograft rejection, inflammatory response, IL6 JAK STAT3 signaling, and IL2 STAT5 signaling. Additionally, SLC2A3 was enriched in the EMT pathway, which is closely linked to tumor development and metastasis. On the other hand, the low-expression group of SLC2A3 was enriched in oxidative phosphorylation, indicating its association with cell metabolism (Fig. **5e**). These findings suggested that the upregulation of SLC2A3 may shift the TME from a metabolic-based state to an immune-response-based state. In the TME, CD8+ T cells play a crucial role in the survival prognosis of HNSC patients, and SLC2A3 may be involved in the negative regulation of CD8+ T cells through various immune response pathways, ultimately determining patient survival prognosis.

## 3.6 High Expression of SLC2A3 May Cause Immune Escape and Immune-suppressive Regulation

**Fig. (6):**
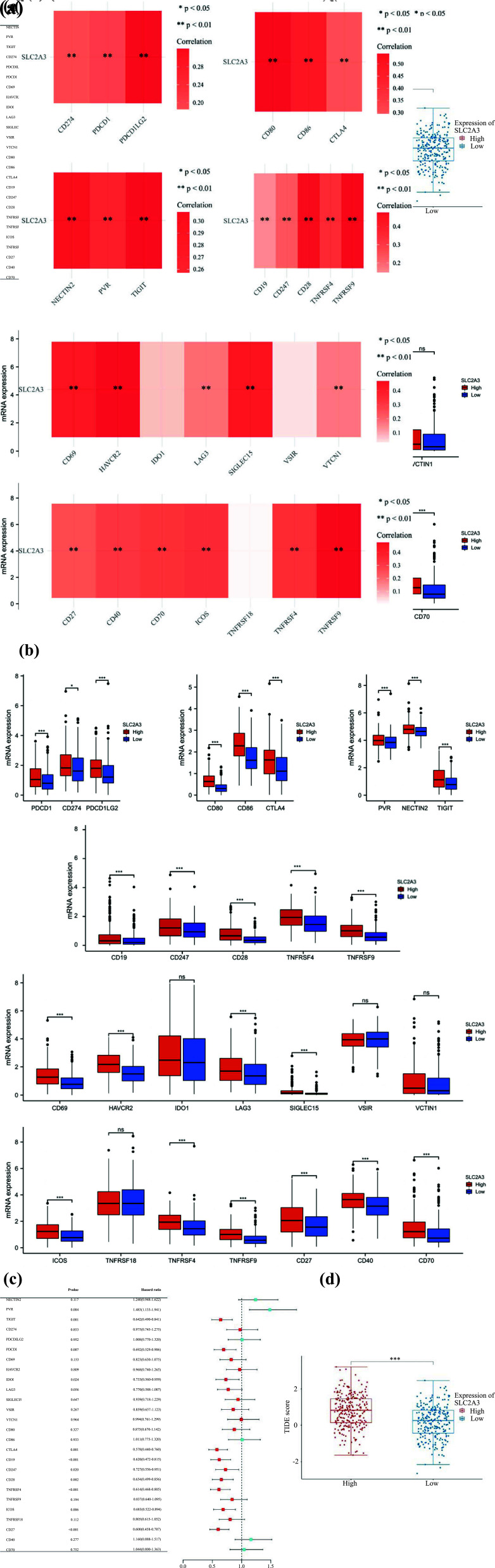
(**a**) Correlation analysis between SLC2A3 expression and types of checkpoints in 504 HNSC cases. (**b**) Comparison of SLC2A3 high- and low-expression group with different types of checkpoints. (**c**) Forest plot of checkpoints. (**d**) box plot of TIDE assays with SLC2A3 high- and low-expression group. Significance levels were denoted as * for *p* < 0.05, ** for *p* < 0.01, *** for *p* < 0.001, and ns for no significance.

The presence of immune checkpoint molecules can impede immune cell function, preventing the body from mounting an effective anti-tumor immune response and allowing the tumor to evade immune surveillance. In 504 HNSC cases, we observed a strong positive correlation between the expression of immune checkpoint targets and SLC2A3 (Fig. **6a**). Furthermore, these checkpoints were significantly upregulated in the SLC2A3 high-expression group (Fig. **6b**), with only 11 of them significantly impacting survival (Fig. **6c**). This suggested that SLC2A3 may promote the expression of immune checkpoints in the tumor microenvironment, leading to a more robust immune suppression and an increased likelihood of immune escape. It is worth noting that the expression of some of these checkpoints can even dictate patient survival.

The TIDE score demonstrated that the SLC2A3 high-expression group exhibited a higher TIDE score (Fig. **6d**). This indicates that high expression of SLC2A3 is associated with two immune escape mechanisms: dysfunction of tumor-infiltrating cytotoxic T lymphocytes (CTLs) and CTL rejection by immunosuppressive factors. Additionally, higher expression of SLC2A3 corresponded to decreased efficacy of immune checkpoint blockade and shortened post-treatment survival.

## 3.7 GEO Validation of Immune Characteristics

**Fig. (7):**
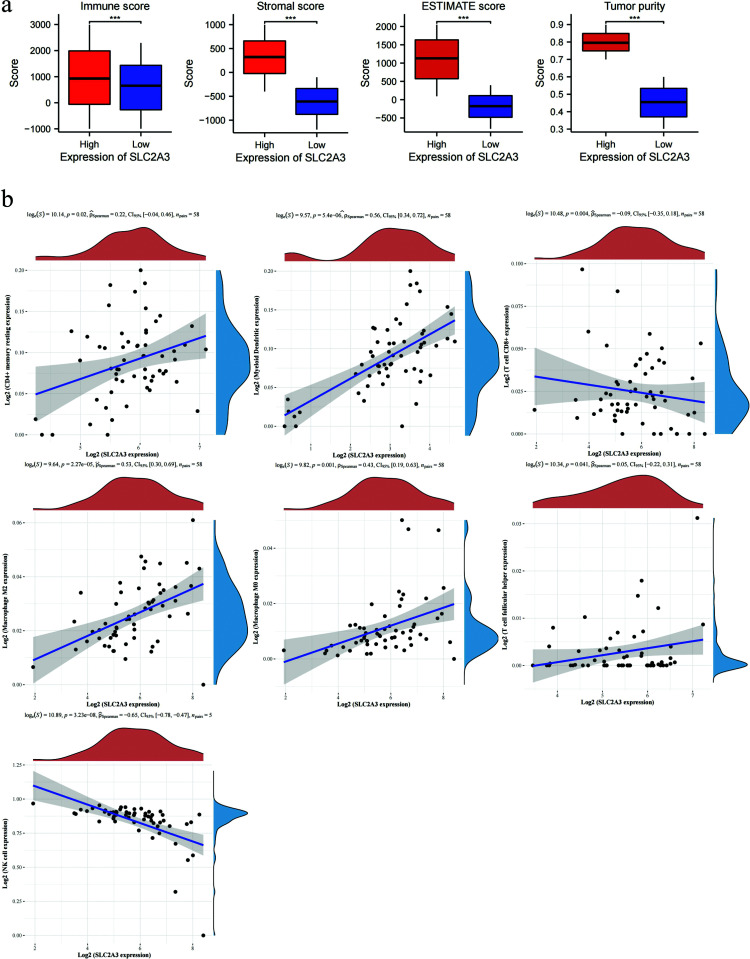
(**a**) ESTIMATE assays obtained immune, stromal, ESTIMATE score and tumor purity. (**b**) EPIC algorithm was used to analyse the correlation between SLC2A3 and immune cells. Significance levels were denoted as * for *p* < 0.05, ** for *p* < 0.01, *** for *p* < 0.001, and ns for no significance.

To validate our findings from the TCGA dataset, we applied the same methodology to a separate dataset (GSE25099) consisting of 57 HNSC samples. Following the same clustering approach, we observed that the immune infiltration (ESTIMATE) in GSE25099 showed a similar pattern to that in the TCGA dataset (Fig. **7a**). We further utilized the EPIC algorithm to examine the correlation between SLC2A3 expression and immune cell subtypes obtained through the intersection of ssGSEA and CIBERSORT analyses. These subtypes included CD4+ memory resting cells, Myeloid Dendritic cells, Macrophage M0, Macrophage M2, T cell follicular helper cells, NK cells, and CD8+ T cells. Interestingly, the results from GSE25099 mirrored the same trend as observed in the TCGA dataset (Fig. **7b**).

## 3.8 Verification of Expression Levels of SLC2A3 in HNSC and Adjacent Tissues

**Fig. (8):**
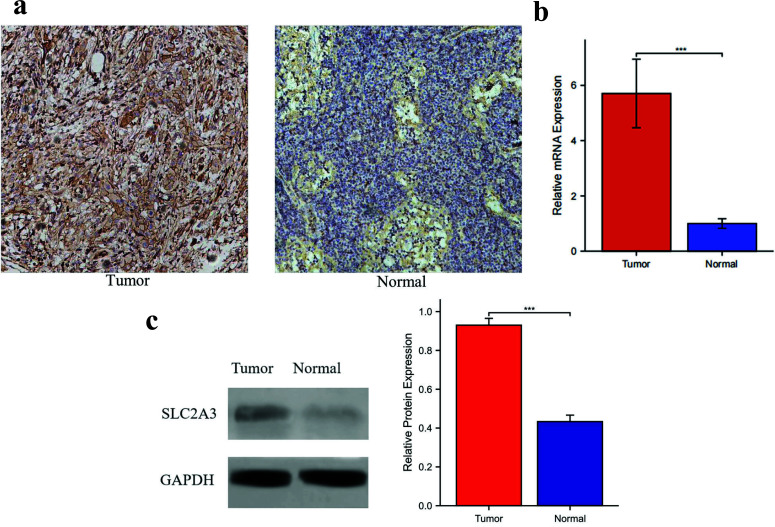
(**a**) Immunohistochemistry staining was used to determine the expression of SLC2A3 in tumor tissues and adjacent tissues. Magnifications ×400. (**b**) SLC2A3 mRNA level was determined by PCR in HNSC tissues and adjacent tissues. (**c**) Western blot was used to assay the protein expression level of SLC2A3 in HNSC tissues and adjacent tissues. Significance levels were denoted as * for *p* < 0.05, ** for *p* < 0.01, *** for *p* < 0.001, and ns for no significance.

To assess the expression levels of the gene SLC2A3, we performed various tests, including PCR, western blot, and IHC, on ten samples of HNSC tissues and their paired adjacent tissues. Our results demonstrated that the expression of SLC2A3 was significantly higher in the HNSC tissues compared to the paired adjacent tissues in both mRNA and protein levels (Fig. **8**).

## 4 DISCUSSION

In our study, we constructed methodologies to measure the complete immune environment in HNSC tumors. Our findings demonstrate that ESTIMATE is an effective process for assessing the immune microenvironment and serves as an effective prognostic marker. The correlation of Immune and Stromal scores with varying clinical prognoses determined that an increasingly complex tumor microenvironment had a significant link to a poorer clinical prognosis. It was also found that a higher level of immune cell infiltration led to a worse clinical stage, tumor grade, and survival probability of HNSC patient. Furthermore, a burgeoning body of evidence states the crucial part immune cells play in the progression of tumors [7, 41-46].

Numerous clinical studies indicated that HNSC is among the tumors characterized by high levels of immune cell infiltration. Factors such as the extent of infiltration of CD8+ T cells and macrophage cells significantly impact its clinical prognosis and overall survival [47-52]. In TIMER, the expression of CD8+ T cells in HNSC was identified as an independent factor that influences the prognosis of patient survival. Daniela Bruni's research also revealed that a high expression of CD8+ T cells in the tumor microenvironment led to a favorable prognosis in 17 solid tumors, especially HNSC, in which the most notable impact of CD8+ T cells on survival was observed in a population of over 1000 HNSC cases [7]. In tumor tissue, CD8+ T cells are commonly regarded as a homogeneous group of cells that secrete significant amounts of cytokines. These factors collaborate to eliminate tumor cells and cells harboring intracellular pathogens [53-61]. Regarding their functionality, CD8+ T cells exert their effects on tumor cells through the elevated expression of perforin, granzyme B, IFN-γ, and TNF-α. Notably, among these factors, IFN-γ collaboratively upregulates major histocompatibility complex class I (MHC-I) on antigen-presenting cells (APCs) in the presence of activation signals [62, 63]. This synergistic action enhances antigen presentation and facilitates the activation of naive T cells in both tumor sites and lymph nodes [64, 65]. Furthermore, TNF-α can potentially contribute to the re-regulation of immunosuppressive cells within the TME, including Tregs, thereby reducing the mechanisms of tumor immune evasion [66-68]. Additionally, Wang *et al.* reported that, apart from the recognized mechanisms, CD8+ T cells can inhibit tumor growth by inducing ferroptosis, providing the initial direct evidence of a connection between ferroptosis and antitumor immunity [69].

Various molecules with dysregulated expression have been identified within the TME of HNSC that are crucial in determining the biological characteristics of tumor cells and the immune response against them [70-72]. In the current study, it was observed that elevated levels of SLC2A3 disrupted the immune microenvironment's equilibrium, leading to an adverse clinical prognosis. Previous research has also demonstrated that increased SLC2A3 expression is associated with worse clinical outcomes [73-75]. Gao *et al.* discovered that SLC2A3 served as an unfavorable prognostic marker in colorectal cancer patients, and its expression was significantly linked to perineural invasion. Previous research has also indicated that elevated expression of SLC2A3, observed in both colorectal and non-small cell lung cancer, acts as a promoter of tumor development [73, 75]. Ayala *et al.* demonstrated that SLC2A3 significantly promotes the aggressiveness and proliferation of oral cancer cells, ultimately leading to a worse clinical prognosis [76, 77]. In terms of tumor metastasis, high SLC2A3 expression was primarily associated with enrichment in the EMT, which is crucial for enhancing tumor cell aggressiveness and metastasis. SLC2A3 exhibited a strong correlation with inflammatory gene expression, and its upregulation induces an inflammatory microenvironment through CXCL8-mediated activation of tumor-associated macrophages, subsequently promoting tumor cell progression and metastasis [78]. In colorectal cancer cells, SLC2A3 expression is regulated by the TGF-β/JNK/ATF2 signaling pathway, thus, increased SLC2A3 levels exacerbate the aggressiveness of colorectal cancer cells [75]. Lactate generated by SLC2A3-mediated glycolysis upregulates IL-6, TGF-β, HGF, and PORCN extracellularly. These factors lead to the activation of β-catenin and STAT3 intracellularly, resulting in the downregulation of E- cadherin and upregulation of N-cadherin. It is worth mentioning that an enhancer located in the second intron of SLC2A3 plays a critical role in responding to various transcription factor activities, and transcription factors such as β- catenin, CTNNB1, and ZEB1 directly bind and activate this enhancer during EMT [79]. Furthermore, within the TME, SLC2A3 is expressed in platelets and is transferred from α- granules to the plasma membrane during degranulation. This process leads to increased glucose uptake and glycolysis in platelets. Additionally, TGF-β is released during degranulation, triggering Smad and NF-κB responses in tumor cells and promoting EMT and metastasis formation [80]. The aerobic glycolysis driven by SLC2A3 not only enhances tumor cell aggressiveness but also initiates a feedback loop that contributes to glucose deficiency in the tumor microenvironment, thereby regulating the inflammatory state of the TME. Under conditions of low glucose, the AMPK/CREB1 signaling pathway is activated in tumor cells, resulting in upregulation of SLC2A3 and creating a positive feedback regulatory loop [75].

Within the tumor microenvironment of HNSC, several molecules with aberrant expression have been shown to influence the biological behavior of tumor cells and the immune response to tumors [71-75, 79, 81]. In the current study, we computed SLC2A3, a cancer promoter that may aggravate TME dysregulation, especially the negative correlation with CD8+T cells. This study highlights a novel mechanism by which SLC2A3 may raise the chaos of the microenvironment to facilitate cancer progression, paving the way for further research. However, the regulatory relationship between SLC2A3 and CD8+ T cells remains unknown and requires further investigation [28]. Clinically, we underpin SLC2A3 as a prognostic marker in HNSC, proposing it as a biomarker for clinical outcomes and offering a potential pathway for predicting disease progression and precision medicine.

## CONCLUSION

Conclusively, we screened SLC2A3, a hub gene associated with tumor immune microenvironment alteration and prognostic values, using immunological algorithms and clinicopathological characteristics. We demonstrated that the high expression of SLC2A3 caused an altered state of the immune microenvironment, enrichment of immune-related biological processes, immune infiltration, and immune escape. Therefore, further experimental validation should be conducted to clarify the mechanism of the effect of SLC2A3 on immune cells and the development of head and neck carcinoma. Together, these findings suggest that SLC2A3 could be used as a biomarker for HNSC prognosis and as a treatment target.

## AUTHORS’ CONTRIBUTIONS

WJ and PL contributed to the conceptualization. WJ contributed to data curation. PL contributed to Funding acquisition. WJ and PL contributed to the methodology. PL contributed to project administration. WJ designed the software. SX was involved in the visualization of the study. WJ wrote the original draft. PL, and SX helped in writing the review and editing of the article.

## Figures and Tables

**Fig. (1):**
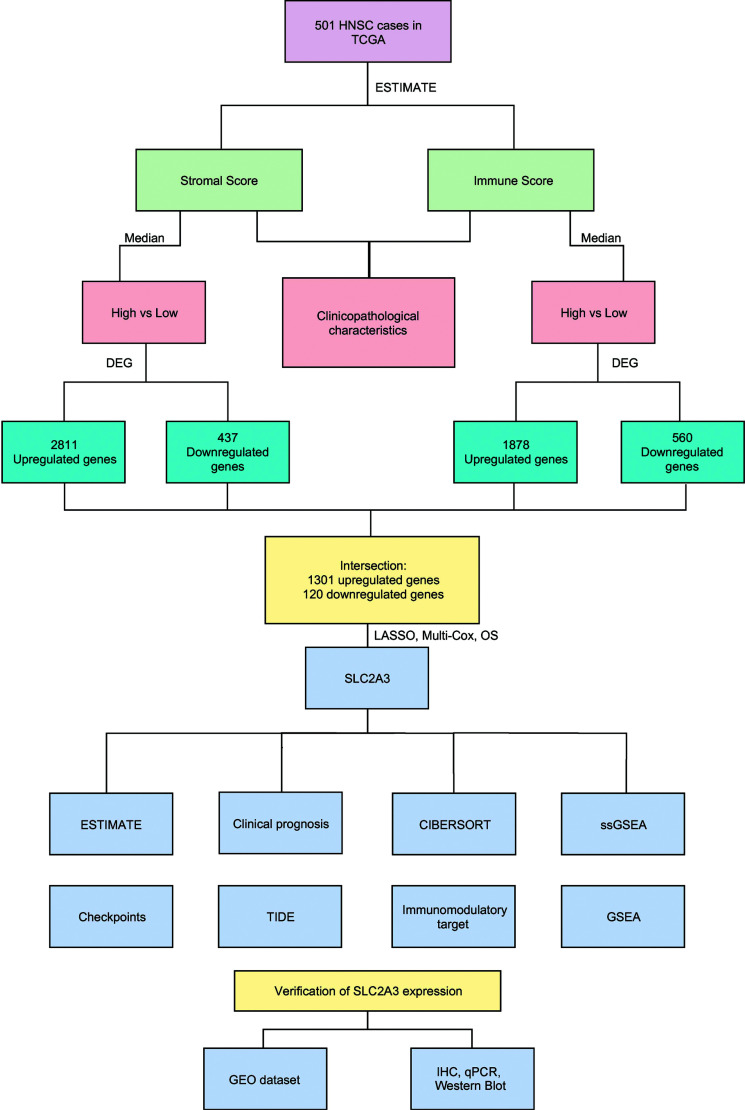
Analysis process of this study.

**Table 1:** The relationship between clinicopathological characters and stromal/immune score.

**Variable**	**No. (%)**	**Stromal Score**			**Immune Score**		
		**M**	**SD**	***P*-value**	**M**	**SD**	***P*-value**
Survival_status	-	-	-	-	-	-	-
Alive	285 (56.6%)	-240.684	714.158	0.453	956.50	771.465	0.061
Dead	219 (43.4%)	-191.386	737.547		824.30	790.213	
Stage	-	-	-	-	-	-	-
I-II	198 (39.2%)	-251.18	703.98	0.425	943.74	801.79	0.008**
III-IV	306 (60.8%)	-198.72	737.15	-	870.36	768.30	-
T stage	-	-	-	-	-	-	-
T1-T2	190 (37.9%)	-221.48	754.08	0.959	1003.057	837.34	<0.001***
T3-T4	314 (62.3%)	-217.96	706.61		836.19	740.33	
N stage	-	-	-	-	-	-	-
N0-N1	324 (64.3%)	-209.01	724.37	0.670	843.79	772.64	0.034*
N2-N3	180 (35.7%)	-237.87	725.20	-	999.23	789.97	-
M stage	-	-	-	-	-	-	-
M0	477 (95.0%)	-202.083	721.633	<0.001***	908.38	780.57	0.267
M1	27 (5.0%)	-546.163	706.152	-	723.31	796.70	-
Grade	-	-	-	-	-	-	-
I-II	362 (72.0%)	-218.421	716.814	0.966	1057.11	736.287	<0.001***
III-IV	142 (28.0%)	-221.515	745.035		837.69	870.735	
Lymphovascular invasion	-	-	-	-	-	-	-
Yes	221 (43.8%)	-238.11	732.13	0.609	875.041	817.916	0.548
No	283 (56.2%)	-204.61	718.70		917.892	753.076	
Perineural invasion	-	-	-	-	-	-	-
Yes	253 (50.2%)	-174.682	736.814	0.167	850.039	751.02	0.159
No	251 (49.8%)	-264.245	709.646		948.628	809.79	

**Note:** Significance levels were denoted as * for *p* < 0.05, ** for *p* < 0.01, and *** for *p* < 0.001.

**Table 2:** The relationship between clinicopathological characters and mRNA expression of SLC2A3.

**Variable**	**No. (%)**	**mRNA Expression of SLC2A3**		
		**M**	**SD**	***P*-value**
Survival_status	-	-	-	-
Alive	285 (56.6%)	2.2544	0.89216	<0.001***
Dead	219 (43.4%)	2.5862	1.10916	
Stage	-	-	-	-
I-II	198 (39.2%)	2.2475	0.89959	0.007**
III-IV	306 (60.8%)	2.4957	1.05705	
T stage	-	-	-	-
T1-T2	190 (37.6%)	2.2557	0.92317	0.011*
T3-T4	314 (62.4%)	2.4844	1.03800	
N stage	-	-	-	-
N0-N1	324 (64.4%)	2.3036	0.91246	0.004**
N2-N3	180 (35.6%)	2.5700	1.13573	
M stage	-	-	-	-
M0	477 (95.0%)	2.4101	0.99713	0.323
M1	27 (5.0%)	2.1761	1.13882	
Grade	-	-	-	-
I-II	362 (72.0%)	2.2978	1.06972	0.008**
III-IV	142 (28.0%)	2.5209	0.96876	
Lymphovascular invasion	-	-	-	-
Yes	221 (43.8%)	2.4005	1.07233	0.968
No	283 (56.2%)	2.3968	0.95062	
Perineural invasion	-	-	-	-
Yes	253 (50.2%)	2.4162	1.04783	0.691
No	251 (49.8%)	2.3805	0.96106	

**Note:** Significance levels were denoted as * for *p* < 0.05, ** for *p* < 0.01, and *** for *p* < 0.001.

## Data Availability

The data that support the findings of this study are available from the corresponding author upon reasonable request. All datasets in this study were downloaded from public databases, permitting their use in scientific research. The tables and figures are sourced from databases that are either in the public domain or permitted under Creative Commons licenses, ensuring they are free of copyright issues.

## LIST OF ABBREVIATIONS

APCs: Antigen-presenting Cells
CTLs: Cytotoxic T Lymphocytes
DEG: Differentially Expressed Genes
EMT: Epithelial-mesenchymal Transition
FDR: False Discovery Rate
GEO: Gene Expression Omnibus
HNSC: Head and Neck Squamous Cell Carcinoma
MHC-I: Major Histocompatibility Complex Class I
TAMs: Tumor-associated Macrophages
TME: Tumor Microenvironment

## References

[1] Bray F., Ferlay J., Soerjomataram I., Siegel R.L., Torre L.A., Jemal A. (2018). Global cancer statistics 2018: GLOBOCAN estimates of incidence and mortality worldwide for 36 cancers in 185 countries.. CA Cancer J. Clin..

[2] Wondergem N.E., Nauta I.H., Muijlwijk T., Leemans C.R., van de Ven R. (2020). The immune microenvironment in head and neck squamous cell carcinoma: On subsets and subsites.. Curr. Oncol. Rep..

[3] Wood S.L., Pernemalm M., Crosbie P.A., Whetton A.D. (2014). The role of the tumor-microenvironment in lung cancer-metastasis and its relationship to potential therapeutic targets.. Cancer Treat. Rev..

[4] Xiao Y., Yu D. (2021). Tumor microenvironment as a therapeutic target in cancer.. Pharmacol. Ther..

[5] Pitt J.M., Marabelle A., Eggermont A., Soria J.C., Kroemer G., Zitvogel L. (2016). Targeting the tumor microenvironment: Removing obstruction to anticancer immune responses and immunotherapy.. Ann. Oncol..

[6] Quail D.F., Joyce J.A. (2013). Microenvironmental regulation of tumor progression and metastasis.. Nat. Med..

[7] Bruni D., Angell H.K., Galon J. (2020). The immune contexture and immunoscore in cancer prognosis and therapeutic efficacy.. Nat. Rev. Cancer.

[8] St Paul M., Ohashi P.S. (2020). The roles of CD8^+^ T cell subsets in antitumor immunity.. Trends Cell Biol..

[9] Rahim M.K., Okholm T.L.H., Jones K.B., McCarthy E.E., Liu C.C., Yee J.L., Tamaki S.J., Marquez D.M., Tenvooren I., Wai K., Cheung A., Davidson B.R., Johri V., Samad B., O’Gorman W.E., Krummel M.F., van Zante A., Combes A.J., Angelo M., Fong L., Algazi A.P., Ha P., Spitzer M.H. (2023). Dynamic CD8^+^ T cell responses to cancer immunotherapy in human regional lymph nodes are disrupted in metastatic lymph nodes.. Cell.

[10] Reina-Campos M., Scharping N.E., Goldrath A.W. (2021). CD8^+^ T cell metabolism in infection and cancer.. Nat. Rev. Immunol..

[11] Feng Z., Bethmann D., Kappler M., Merino B.C., Eckert A., Bell R.B., Cheng A., Bui T., Leidner R., Urba W.J., Johnson K., Hoyt C., Bifulco C.B., Bukur J., Wickenhauser C., Seliger B., Fox B.A. (2017). Multiparametric immune profiling in HPV– oral squamous cell cancer.. JCI Insight.

[12] Eberhardt C.S., Kissick H.T., Patel M.R., Cardenas M.A., Prokhnevska N., Obeng R.C., Nasti T.H., Griffith C.C., Im S.J., Wang X., Shin D.M., Carrington M., Chen Z.G., Sidney J., Sette A., Saba N.F., Wieland A., Ahmed R. (2021). Functional HPV-specific PD-1^+^ stem-like CD8 T cells in head and neck cancer.. Nature.

[13] Yoshihara K., Shahmoradgoli M., Martínez E., Vegesna R., Kim H., Garcia T.W., Treviño V., Shen H., Laird P.W., Levine D.A., Carter S.L., Getz G., Stemke-Hale K., Mills G.B., Verhaak R.G.W. (2013). Inferring tumour purity and stromal and immune cell admixture from expression data.. Nat. Commun..

[14] Wong R.S.Y. (2011). Apoptosis in cancer: From pathogenesis to treatment.. J. Exp. Clin. Cancer Res..

[15] Noy R., Pollard J.W. (2014). Tumor-associated macrophages: From mechanisms to therapy.. Immunity.

[16] Bader J.E., Voss K., Rathmell J.C. (2020). Targeting metabolism to improve the tumor microenvironment for cancer immunotherapy.. Mol. Cell.

[17] De Palma M., Lewis C.E. (2013). Macrophage regulation of tumor responses to anticancer therapies.. Cancer Cell.

[18] Gunassekaran G.R., Vadevoo P.S.M., Baek M.C., Lee B. (2021). M1 macrophage exosomes engineered to foster M1 polarization and target the IL-4 receptor inhibit tumor growth by reprogramming tumor-associated macrophages into M1-like macrophages.. Biomaterials.

[19] Gao J., Liang Y., Wang L. (2022). Shaping polarization of tumor-associated macrophages in cancer immunotherapy.. Front. Immunol..

[20] Folkman J. (2003). Angiogenesis and apoptosis.. Semin. Cancer Biol..

[21] Tong X., Tang R., Xiao M., Xu J., Wang W., Zhang B., Liu J., Yu X., Shi S. (2022). Targeting cell death pathways for cancer therapy: Recent developments in necroptosis, pyroptosis, ferroptosis, and cuproptosis research.. J. Hematol. Oncol..

[22] Arner E.N., Rathmell J.C. (2023). Metabolic programming and immune suppression in the tumor microenvironment.. Cancer Cell.

[23] Chen Y., Song Y., Du W., Gong L., Chang H., Zou Z. (2019). Tumor-associated macrophages: An accomplice in solid tumor progression.. J. Biomed. Sci..

[24] Xia L., Oyang L., Lin J., Tan S., Han Y., Wu N., Yi P., Tang L., Pan Q., Rao S., Liang J., Tang Y., Su M., Luo X., Yang Y., Shi Y., Wang H., Zhou Y., Liao Q. (2021). The cancer metabolic reprogramming and immune response.. Mol. Cancer.

[25] Chen C., Wang Z., Ding Y., Qin Y. (2023). Tumor microenvironment-mediated immune evasion in hepatocellular carcinoma.. Front. Immunol..

[26] Alonso M.H., Aussó S., Doriga L.A., Cordero D., Guinó E., Solé X., Barenys M., de Oca J., Capella G., Salazar R., Pamplona S.R., Moreno V. (2017). Comprehensive analysis of copy number aberrations in microsatellite stable colon cancer in view of stromal component.. Br. J. Cancer.

[27] Li T., Fu J., Zeng Z., Cohen D., Li J., Chen Q., Li B., Liu X.S. (2020). TIMER2.0 for analysis of tumor-infiltrating immune cells.. Nucleic Acids Res..

[28] Xiang S., Li J., Shen J., Zhao Y., Wu X., Li M., Yang X., Kaboli P.J., Du F., Zheng Y., Wen Q., Cho C.H., Yi T., Xiao Z. (2021). Identification of prognostic genes in the tumor microenvironment of hepatocellular carcinoma.. Front. Immunol..

[29] Zheng X., Ma Y., Bai Y., Huang T., Lv X., Deng J., Wang Z., Lian W., Tong Y., Zhang X., Yue M., Zhang Y., Li L., Peng M. (2022). Identification and validation of immunotherapy for four novel clusters of colorectal cancer based on the tumor microenvironment.. Front. Immunol..

[30] Fane M., Weeraratna A.T. (2020). How the ageing microenvironment influences tumour progression.. Nat. Rev. Cancer.

[31] Cheng P., Ma J., Zheng X., Zhou C., Chen X. (2021). Bioinformatic profiling identifies prognosis-related genes in the immune microenvironment of endometrial carcinoma.. Sci. Rep..

[32] Hanahan D., Weinberg R.A. (2011). Hallmarks of cancer: The next generation.. Cell.

[33] Ziegler G.C., Almos P., McNeill R.V., Jansch C., Lesch K.P. (2020). Cellular effects and clinical implications of *SLC2A3* copy number variation.. J. Cell. Physiol..

[34] Xiang J., Chen H., Lin Z., Chen J., Luo L. (2023). Identification and experimental validation of ferroptosis-related gene *SLC2A3* is involved in rheumatoid arthritis.. Eur. J. Pharmacol..

[35] Lin L., Que R., Wang J., Zhu Y., Liu X., Xu R. (2022). Prognostic value of the ferroptosis-related gene *SLC2A3* in gastric cancer and related immune mechanisms.. Front. Genet..

[36] Lang F., Singh Y., Salker M.S., Ma K., Pandyra A.A., Lang P.A., Lang K.S. (2020). Glucose transport in lymphocytes.. Pflugers Arch..

[37] Estilo CL (2019). Oral tongue cancer gene expression profiling: Identification of novel potential prognosticators by oligonucleotide microarray analysis.. BMC Cancer.

[38] Song M.Y., Lee D.Y., Yun S.M., Kim E.H. (2022). GLUT3 promotes epithelial–mesenchymal transition *via* TGF-β/JNK/ATF2 signaling pathway in colorectal cancer cells.. Biomedicines.

[39] Yao X., He Z., Qin C., Deng X., Bai L., Li G., Shi J. (2020). SLC2A3 promotes macrophage infiltration by glycolysis reprogramming in gastric cancer.. Cancer Cell Int..

[40] Lavoro A., Falzone L., Tomasello B., Conti G.N., Libra M., Candido S. (2023). *In silico* analysis of the solute carrier (SLC) family in cancer indicates a link among DNA methylation, metabolic adaptation, drug response, and immune reactivity.. Front. Pharmacol..

[41] Chan T.A., Yarchoan M., Jaffee E., Swanton C., Quezada S.A., Stenzinger A., Peters S. (2019). Development of tumor mutation burden as an immunotherapy biomarker: Utility for the oncology clinic.. Ann. Oncol..

[42] Zhang X., Shi M., Chen T., Zhang B. (2020). Characterization of the immune cell infiltration landscape in head and neck squamous cell carcinoma to aid immunotherapy.. Mol. Ther. Nucleic Acids.

[43] McGrail D.J., Pilié P.G., Rashid N.U., Voorwerk L., Slagter M., Kok M., Jonasch E., Khasraw M., Heimberger A.B., Lim B., Ueno N.T., Litton J.K., Ferrarotto R., Chang J.T., Moulder S.L., Lin S.Y. (2021). High tumor mutation burden fails to predict immune checkpoint blockade response across all cancer types.. Ann. Oncol..

[44] Kiely M., Lord B., Ambs S. (2022). Immune response and inflammation in cancer health disparities.. Trends Cancer.

[45] Darvin P., Toor S.M., Nair S.V., Elkord E. (2018). Immune checkpoint inhibitors: Recent progress and potential biomarkers.. Exp. Mol. Med..

[46] Hoekstra M.E., Vijver S.V., Schumacher T.N. (2021). Modulation of the tumor micro-environment by CD8+ T cell-derived cytokines.. Curr. Opin. Immunol..

[47] Park J., Hsueh P.C., Li Z., Ho P.C. (2023). Microenvironment-driven metabolic adaptations guiding CD8^+^ T cell anti-tumor immunity.. Immunity.

[48] Philip M., Schietinger A. (2022). CD8^+^ T cell differentiation and dysfunction in cancer.. Nat. Rev. Immunol..

[49] Han J., Khatwani N., Searles T.G., Turk M.J., Angeles C.V. (2020). Memory CD8^+^ T cell responses to cancer.. Semin. Immunol..

[50] Gajewski T.F., Schreiber H., Fu Y.X. (2013). Innate and adaptive immune cells in the tumor microenvironment.. Nat. Immunol..

[51] Farhood B., Najafi M., Mortezaee K. (2019). CD8 ^+^ cytotoxic T lymphocytes in cancer immunotherapy: A review.. J. Cell. Physiol..

[52] van der Leun A.M., Thommen D.S., Schumacher T.N. (2020). CD8^+^ T cell states in human cancer: Insights from single-cell analysis.. Nat. Rev. Cancer.

[53] Lei X., Lei Y., Li J.K., Du W.X., Li R.G., Yang J., Li J., Li F., Tan H.B. (2020). Immune cells within the tumor microenvironment: Biological functions and roles in cancer immunotherapy.. Cancer Lett..

[54] Schreiber R.D., Old L.J., Smyth M.J. (2011). Cancer immunoediting: Integrating immunity’s roles in cancer suppression and promotion.. Science.

[55] Maiorino L., Daßler-Plenker J., Sun L., Egeblad M. (2022). Innate immunity and cancer pathophysiology.. Annu. Rev. Pathol..

[56] Mao X., Xu J., Wang W., Liang C., Hua J., Liu J., Zhang B., Meng Q., Yu X., Shi S. (2021). Crosstalk between cancer-associated fibroblasts and immune cells in the tumor microenvironment: New findings and future perspectives.. Mol. Cancer.

[57] Cao L.L., Kagan J.C. (2023). Targeting innate immune pathways for cancer immunotherapy.. Immunity.

[58] Zaidi M.R., Merlino G. (2011). The two faces of interferon-γ in cancer.. Clin. Cancer Res..

[59] Burke J.D., Young H.A. (2019). IFN-γ: A cytokine at the right time, is in the right place.. Semin. Immunol..

[60] Dhatchinamoorthy K., Colbert J.D., Rock K.L. (2021). Cancer immune evasion through loss of MHC class I antigen presentation.. Front. Immunol..

[61] Sari G., Rock K.L. (2023). Tumor immune evasion through loss of MHC class-I antigen presentation.. Curr. Opin. Immunol..

[62] Overacre-Delgoffe A.E., Chikina M., Dadey R.E., Yano H., Brunazzi E.A., Shayan G., Horne W., Moskovitz J.M., Kolls J.K., Sander C., Shuai Y., Normolle D.P., Kirkwood J.M., Ferris R.L., Delgoffe G.M., Bruno T.C., Workman C.J., Vignali D.A.A. (2017). Interferon-γ drives Treg fragility to promote anti-tumor immunity.. Cell.

[63] Li D., Li W., Zheng P., Yang Y., Liu Q., Hu Y., He J., Long Q., Ma Y. (2023). A “trained immunity” inducer-adjuvanted nanovaccine reverses the growth of established tumors in mice.. J. Nanobiotechnology.

[64] Castro F., Cardoso A.P., Gonçalves R.M., Serre K., Oliveira M.J. (2018). Interferon-gamma at the crossroads of tumor immune surveillance or evasion.. Front. Immunol..

[65] Gocher A.M., Workman C.J., Vignali D.A.A. (2022). Interferon-γ: Teammate or opponent in the tumour microenvironment?. Nat. Rev. Immunol..

[66] Mehta A.K., Gracias D.T., Croft M. (2018). TNF activity and T cells.. Cytokine.

[67] Balkwill F. (2006). TNF-α in promotion and progression of cancer.. Cancer Metastasis Rev..

[68] Cruceriu D., Baldasici O., Balacescu O., Neagoe B.I. (2020). The dual role of tumor necrosis factor-alpha (TNF-α) in breast cancer: molecular insights and therapeutic approaches.. Cell Oncol..

[69] Wang W., Green M., Choi J.E., Gijón M., Kennedy P.D., Johnson J.K., Liao P., Lang X., Kryczek I., Sell A., Xia H., Zhou J., Li G., Li J., Li W., Wei S., Vatan L., Zhang H., Szeliga W., Gu W., Liu R., Lawrence T.S., Lamb C., Tanno Y., Cieslik M., Stone E., Georgiou G., Chan T.A., Chinnaiyan A., Zou W. (2019). CD8^+^ T cells regulate tumour ferroptosis during cancer immunotherapy.. Nature.

[70] Barron C.C., Bilan P.J., Tsakiridis T., Tsiani E. (2016). Facilitative glucose transporters: Implications for cancer detection, prognosis and treatment.. Metabolism.

[71] Ruffin A.T., Li H., Vujanovic L., Zandberg D.P., Ferris R.L., Bruno T.C. (2023). Improving head and neck cancer therapies by immunomodulation of the tumour microenvironment.. Nat. Rev. Cancer.

[72] Chen S.M.Y., Krinsky A.L., Woolaver R.A., Wang X., Chen Z., Wang J.H. (2020). Tumor immune microenvironment in head and neck cancers.. Mol. Carcinog..

[73] Masin M., Vazquez J., Rossi S., Groeneveld S., Samson N., Schwalie P.C., Deplancke B., Frawley L.E., Gouttenoire J., Moradpour D., Oliver T.G., Meylan E. (2014). GLUT3 is induced during epithelial-mesenchymal transition and promotes tumor cell proliferation in non-small cell lung cancer.. Cancer Metab..

[74] Gökalp F. (2022). An investigation into the usage of monosaccharides with GLUT1 and GLUT3 as prognostic indicators for cancer.. Nutr. Cancer.

[75] Dai W., Xu Y., Mo S., Li Q., Yu J., Wang R., Ma Y., Ni Y., Xiang W., Han L., Zhang L., Cai S., Qin J., Chen W.L., Jia W., Cai G. (2020). GLUT3 induced by AMPK/CREB1 axis is key for withstanding energy stress and augments the efficacy of current colorectal cancer therapies.. Signal Transduct. Target. Ther..

[76] Gao H., Hao Y., Zhou X., Li H., Liu F., Zhu H., Song X., Niu Z., Ni Q., Chen M.S., Lu J. (2019). Prognostic value of glucose transporter 3 expression in hepatocellular carcinoma.. Oncol. Lett..

[77] Ayala F.R.R., Rocha R.M., Carvalho K.C., Carvalho A.L., Da Cunha I.W., Lourenço S.V., Soares F.A. (2010). GLUT1 and GLUT3 as potential prognostic markers for oral squamous cell carcinoma.. Molecules.

[78] Tsai T.H., Yang C.C., Kou T.C., Yang C.E., Dai J.Z., Chen C.L., Lin C.W. (2021). Overexpression of GLUT3 promotes metastasis of triple-negative breast cancer by modulating the inflammatory tumor microenvironment.. J. Cell. Physiol..

[79] Ancey P.B., Contat C., Meylan E. (2018). Glucose transporters in cancer – from tumor cells to the tumor microenvironment.. FEBS J..

[80] Labelle M., Begum S., Hynes R.O. (2011). Direct signaling between platelets and cancer cells induces an epithelial-mesenchymal-like transition and promotes metastasis.. Cancer Cell.

[81] Choi H., Na K.J. (2021). Different glucose metabolic features according to cancer and immune cells in the tumor microenvironment.. Front. Oncol..

