# Supplementary material for: SLC2A3 is a Potential Factor for Head and Neck Squamous Cancer Development through Tumor Microenvironment Alteration
Source: Curr Gene Ther. 2024 May 21;25(2):157–77. doi: 10.2174/0115665232291300240509104344 (PMC11774314; doi:10.2174/0115665232291300240509104344)
Supplement: Supplementary file 1 — Supplementary material is available on the publisher’s website along with the published article. [file CGT-25-2-157_SD1.pdf]

Supplementary Material

SLC2A3 is a Potential Factor for Head and Neck Squamous Cancer Development through Tumor Microenvironment Alteration

Wei Jiang[1,2], Sheng Xu[3,*] and Ping Li[4,*]

[1]Guangxi Key Laboratory of Early Prevention and Treatment for Regional High Frequency Tumor, Guangxi Medical University, Nanning, Guangxi Zhuang Autonomous Region, China; [2]College of Stomatology, Guangxi Medical University, Nanning, Guangxi Zhuang Autonomous Region, China; [3]Department of Dental Laboratory, Guangxi Medical University College of Stomatology, Nanning, Guangxi Zhuang Autonomous Region, China; [4]Department of Pathology, Guangxi Medical University College of Stomatology, Nanning, Guangxi Zhuang Autonomous Region, China

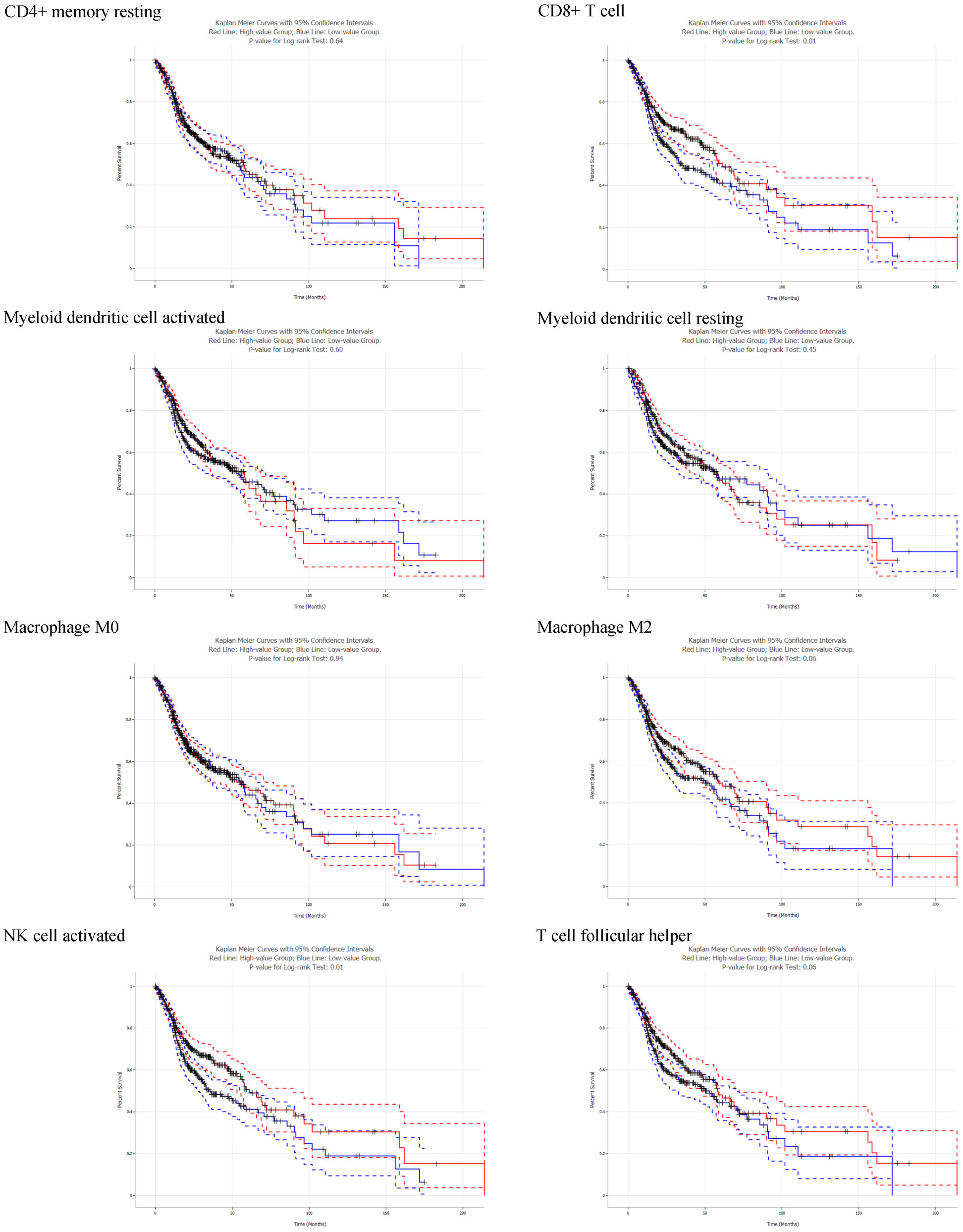

Fig. (S1).
